# A broad-based probe-free qPCR assay for detection and discrimination of three human herpes viruses

**DOI:** 10.1016/j.jviromet.2023.114824

**Published:** 2023-09-29

**Authors:** Anshu Gupta, Shelley M. Lawrence, Stephanie I. Fraley

**Affiliations:** a Jacobs School of Engineering, Department of Bioengineering, University of California San Diego, La Jolla, CA, USA; b Department of Pediatrics, Division of Neonatology, University of Utah, Salt Lake City, UT, USA

**Keywords:** Herpes, CMV, HSV, High resolution melt, Broad-range PCR

## Abstract

Primary infection or reactivation of latent human cytomegalovirus (HCMV) or herpes simplex viruses (HSV) 1 or 2 during pregnancy can transmit the virus *in utero* or during natural childbirth to the fetus. The majority of these infections are asymptomatic at birth but may present later with potentially lethal disseminated infection or meningitis (HSV), or long-term neurodevelopmental sequelae including sensorineural hearing loss or neurodevelopmental impairments (HCMV). Unfortunately, early signs and symptoms of disseminated viral infections may be misdiagnosed as bacterial sepsis. Therefore, immediate testing for viral etiologies may not be ordered or even considered by skilled clinicians. In asymptomatic HCMV infections, early detection is necessary to monitor for and treat future neurologic sequelae. In acutely ill-appearing infants, specific detection of viruses against other disease-causing agents is vital to inform correct patient management, including early administration of the correct antimicrobial(s). An ideal test should be rapid, inexpensive, require low sample volumes, and demonstrate efficacy in multiple tissue matrices to aid in timely clinical decision-making for neonatal infections. This work discusses the development of a rapid probe-free qPCR assay for HSV and HCMV that enables early and specific detection of these viruses in neonates. The assay’s probe free chemistry would allow easier extension to a broad-based multiplexed pathogenic panel as compared to assays utilizing sequence-specific probes or nested PCR.

## 1. Introduction

Human cytomegalovirus is the most common cause of congenital viral infection in humans and the leading non-genetic etiology of neurodevelopmental sequelae in children, including intellectual disability, cerebral palsy, seizures, visual defects and sensorineural hearing loss (Cannon et al., 2014; Ross et al., 2012; Pass, 1985; Davis et al., 2017; Dimech et al., 2018; Kenneson and Cannon, 2007; Lanari et al., 2006; Manicklal et al., 2013). HCMV seroprevalence among women of reproductive age is high, ranging from 45 % to 100 % worldwide (Cannon et al., 2010). In the United States, an estimated 58.3 % of pregnant women harbor the virus (Staras et al., 2006), with an additional 27,000 new CMV infections reported in pregnant women each year (Colugnati et al., 2007). The infection is usually asymptomatic but active viral replication in pregnant women, resulting from new (primary) infections or reinfection/reactivation of latent virus (non-primary infection) can lead to congenital CMV infection or cCMV in the fetus. While the risk for cCMV from latent primary infections is < 3 %, it can range from 30 % to 70 % for primary infection or viral reactivation (Cannon et al., 2010). Therefore, one in every 200 babies born in the US will be infected with cCMV according to CDC data (https://www.cdc.gov/cmv/clinical/congenital-cmv.html), or an estimated 30,000 cases annually.

Although the majority (90 %) of neonates with cCMV infection are asymptomatic at birth, an estimated 1 in 10 of well-appearing cCMV-positive infants will develop late onset health conditions, including sensorineural hearing loss (SNHL), months to years after their initial infection (Nigro, 2009). The remaining 10% of infants infected with cCMV *in utero* will present with clinical symptoms at birth (Nigro, 2009) and nearly half will develop significant long term neurodevelopmental challenges (from CDC, https://www.cdc.gov/cmv/clinical/congenital-cmv.html). Most cases (>50 %) of cCMV resulting in SNHL are progressive in nature and may be missed by standard, routine hearing screens at the time of birth (Manicklal et al., 2013). As symptoms of progressive hearing loss and/or clinical presentation of neurologic disabilities are not specific to HCMV, clinicians may order multiple diagnostic tests and/or ineffective therapies in the quest to diagnose other causes, which can substantially increase medical costs, while failing to identify the actual cause (Cannon et al., 2014). cCMV infections also need to be diagnosed within the first three weeks of life, after which, congenital infections cannot be distinguished from postnatally acquired infections, which are usually not associated with neurologic sequelae (Swanson and Schleiss, 2013).

Rapid, inexpensive, specific and early detection of cCMV is thus required to manage disease progression and inform early intervention. First, earlier CMV diagnosis can help initiate timely therapy and prevent/decrease the development of sequelae, particularly in case of asymptomatic and late onset conditions. Second, cCMV screening would help to monitor future hearing loss and provide timely intervention, which has been known to improve receptive and expressive language skills (Cannon et al., 2014). Third, early diagnosis would incur significant cost savings by avoiding extraneous diagnostic tests or therapies in the quest to find the actual source of CMV caused neurodevelopmental impairments (Cannon et al., 2014). Fourth, a precise and rapid CMV diagnostic test would also aid in antiviral clinical trials by identifying the neonates that may benefit from this therapy.

Like HCMV, herpes simplex viruses (HSV) are types of herpes viruses that are known to infect humans. There are two types of herpes simplex viruses: (a) HSV1 that has been traditionally associated with oral, labial, and facial lesions and (b) HSV2 that is the common etiology of genital herpes. However, recent studies indicate HSV1, a more virulent strain than HSV2, is rapidly rising as a primary etiology of genital herpes, leading to 20–50 % of current cases (Wald and Corey, 2007; Fatahzadeh and Schwartz, 2007). HSV, like HCMV, establishes latency in the host with a particular predilection for neurons of dorsal root ganglia and autonomic nervous system (Whitley and Roizman, 2001).

Seroprevalence of HSV infections in women of ages 15–49 in the US is 50.9 % for HSV1 and 15.9 % for HSV2 based on CDC (https://www.cdc.gov/nchs/products/databriefs/db304.htm). Two in three women are either asymptomatic or show ambiguous symptoms which makes it difficult to diagnose and treat the infection (Whitley et al., 1980; Pinninti and Kimberlin, 2014). HSV can be transmitted from a pregnant woman to the neonate trans-placentally *in utero* in 5 % cases, through contact with infected genital secretions peripartum in 85 % cases or postnatally in 10 % infections (Kimberlin, 2007). Like CMV, transmission risk is higher for primary infections (57 %) as compared to 25 % for non-primary infections and 2% for recurring infections (Pinninti and Kimberlin, 2014). Unlike HCMV, however, HSV transmission does not occur via ingestion of breast milk (James et al., 2014).

Neonatal HSV infection (nHSV) is less common than cCMV, with occurrence in every 1 in 3200 births annually in the US, or around 1500 nHSV cases every year (Pinninti and Kimberlin, 2014; James et al., 2014). nHSV may present as skin-eye-mouth disease (SEM; 45%) such as keratitis and conjunctivitis, disseminated disease (25 %) or neurologic disease (30 %), including meningitis and encephalitis (James et al., 2014; James and Kimberlin, 2015). Without therapy, the mortality rate associated with disseminated disease is 85 % and 50 % for central nervous system (CNS) disease, which is greatly reduced to 31 % and 6 %, respectively, with antiviral therapy (Corey and Wald, 2009). This underscores the need for early diagnosis and timely therapy for nHSV. HSV is also the major cause of viral sepsis and has many common non-specific symptoms with other sepsis causing pathogens. It may, therefore, be difficult to differentiate HSV from more common pathogens that cause neonatal sepsis, particularly in the absence of skin lesions (Lin et al., 2018). The gold standard in sepsis detection is blood culture (Sinha et al., 2018a), which cannot detect HSV and other viral agents.

An early and rapid test for HSV is thus necessary for the following reasons. First, it will allow early diagnosis and intervention of otherwise asymptomatic infections in neonates. Second, it can help specific detection and correct treatment for neonatal sepsis in case of non-specific symptoms, which may otherwise be misdiagnosed or left undiagnosed. Third, it can be used to rapidly screen laboring women for active HSV infection that will inform the safest mode of delivery. In case of infection, cesarean delivery could be ordered to reduce peripartum transmission risk through the birth canal. The newborn can also be rapidly identified as at risk for HSV infection and appropriate postnatal observation and clinical management initiated.

Therefore, the aim of this work was to develop a probe-free universal real time PCR based assay to detect HCMV and HSV1 and 2 through high resolution melt (HRM) technology. The current gold standard for HCMV and HSV1 and 2 diagnosis is viral culture (Hodinka and Kaiser, 2013), which does not satisfy the requirements for a rapid test for early neonatal detection as discussed above. These tests have slower turn-around times that makes them unsuitable as a screening tool, have decreased sensitivity (Razonable et al., 2020), higher costs than molecular tests and require larger sample volumes. Multiplexed molecular tests are also used in some laboratories. At these sites, the diagnostic work-up for suspected neonatal HCMV infection typically includes qPCR from whole blood and urine, while suspected HSV infection involves qPCR from plasma and CSF (Melvin et al., 2015; Forner et al., 2015a). These tests usually utilize universal primers or multiple targeted primers added together in the same reaction, along with either nested PCR (same external but different species-specific internal primers) or sequence specific fluorescent probes to differentiate each species (specificity) (Johnson et al., 2000). This limits the number of pathogens that can be detected in a single reaction chemistry due to a cap on the maximum number of different oligonucleotides (primers or probes) that can be used effectively in the same reaction. Some assays split multiple primers/probes across different reactions, leading to a larger sample volume requirement to maintain assay sensitivity. Studies using such assays have reported that the HCMV load in the blood of infected babies at birth ranges from ~1000 – 50,000 copies/ml, with higher loads correlating with late-onset disease (Forner et al., 2015a). HCMV load in urine ranges from ~4000 – 95,000,000 copies/ml (de Vries et al., 2012). Meanwhile, HSV DNA levels in the plasma of infants have been reported to range from ~1–1,000,000 copies/ml, with higher levels correlating with systemic disease and severity (Melvin et al., 2015). HSV DNA in cerebrospinal fluid (CSF) has been reported to range from ~1–350,000 copies/ml (Melvin et al., 2015).

This work hypothesizes the use of probe-free assays with universal primer chemistry and specificity based on high-resolution melt technology to alleviate the above limitations on broad based pathogen detection. Instead of sequence specific probes, species level identification is accomplished by high resolution melt signature that uses non-specific fluorescent intercalating dyes. The use of probe free assays would also allow extension of the assay to include more pathogens without redesigning new probes for each new species. Here, a universal probe free quantitative PCR (qPCR) assay was designed to detect HCMV, HSV1 and HSV2 utilizing high-resolution melt technology. The work involves primer design from viral sequences, qPCR assay optimization for the primers and its analytical characterization. The assay would help in improving neonatal disease management through early detection and timely intervention, specifically in cases of asymptomatic infections. The inherent rapidity of the qPCR test as compared to viral culture and its higher sensitivity and cost effectiveness also allow it to be used as a screening tool for maternal viral infections during pregnancy or for universal newborn screening for these viruses.

## 2. Materials and methods

### 2.1. Design of primers

To find universal primers for the three human herpesviruses, 6 gene families whose products are evolutionarily conserved in these viruses were considered: (a)capsid, (b)tegument and cytoplasmic egress, (c) envelope, (d)regulation, (e)DNA replication, recombination and metabolism, and (f)capsid assembly, DNA encapsidation and nuclear egress (Mocarski, 2007). Each gene from the six selected gene families was manually aligned for the three viruses (HCMV:NC_006273.2, HSV1: NC_001806.2 and HSV2: NC_001798.2) using the online Benchling alignment tool (http://www.benchling.com) to find all possible primers conserved in the three species. The primers were analyzed for homo-dimerization, hetero-dimerization and hairpin formation *in silico* using online tools: IDT oligo analyzer, Sigma Aldrich OligoEvaluator and PREMIER Biosoft NetPrimer, to obtain 51 primer pairs. To our knowledge, only one of these primers is already found in literature (Supplementary Table 1) (Rozenberg and Lebon, 1991). Barring one primer pair from the Glycoprotein B gene, all the primers are from the highly conserved DNA Polymerase gene of the viruses (Johnson et al., 2000). The primers range in length from 18 to 30 bases.

### 2.2. Human and viral DNA

Human genomic DNA for specificity experiments was extracted from frozen human cord blood samples obtained from Rady Children’s Hospital. The frozen blood was thawed and DNA extracted in multiple aliquots using Promega Wizard Genomic DNA Purification kit using the vendor recommended protocol for whole blood to obtain an average of 160 μg/ml DNA in PCR purified water. Extracted viral DNA was obtained from ATCC with the following product details: HCMV strain AD-169-ATCC VR-538D, HSV1 strain KOS-ATCC VR-1493D and HSV2 strain G-ATCC VR-734.

### 2.3. Testing of primer specificity against human DNA

All 51 primer pairs were tested against human DNA amplification. Each primer set was tested in four reactions, two with no template (no template controls or NTC) and two with extracted human genomic DNA as the template. All reaction volumes were 15 μL with 1x NEB Q5 buffer, 2.5x EvaGreen, 0.2 mM dNTPs, 0.02 U/μL of NEB Q5 Polymerase and 0.5 μM each of forward and reverse primers. Human DNA was used at a final reaction concentration of 3.2 ng/μL. Thermocycling was performed in Thermo Fisher QuantStudio Real-Time PCR System at the following conditions: 1.5 min of initial denaturation at 98° C, 55x cycles of 10 s denaturation at 98° C, 30 s annealing at varying temperatures and 30 s elongation at 72° C, followed by a 5-minute final extension at 72° C. Post the amplification cycles, the reactions were heated to 95° C followed by a melting curve temperature gradient from 60° C to 99.9° C. The annealing temperatures were estimated from NEB Tm calculator (http://www.tmcalculator.neb.com) to be 69° C for primer sets 1–8, 72° C for primer sets 9–22 and 47–51 and 70° C for primer sets 23–46.

Amplification and melt curves for each primer pair were analyzed and gel electrophoreses performed to find primers that do not amplify human DNA. The selected primer pairs were then re-subjected to PCR in 12 reactions-2 as NTCs and 10 with human DNA as the template. Primer sets that did not amplify human DNA were selected for further testing.

### 2.4. Real time PCR assay development for herpes viruses

Selected primer sets that did not amplify human DNA were tested for HCMV (1.53 × 10^5^ genome copies/reaction) and HSV1 (5.23 ×10^5^ genome copies/reaction) amplification in duplicates with 2 separate reactions as NTC. The thermocycling conditions for amplification and melting are the same as followed previously. Analysis was performed on their melt and amplification curves and gel electrophoresis. The primer set showing specific amplification of the two viruses were chosen and retested in qPCR in replicates of 6 for each virus. HSV2 testing was conducted after the designed and selected primer assay was optimized, since HSV2 genome is similar to HSV1. An alignment of this primer set to the target genomes and to closely related VZV, HHV6a, and HHV6b genomes is shown in Supplementary Fig. 1. The selected primers contain multiple mismatches to VZV and HHV6a/b, including in the clamp regions, which indicate that these organisms will not be amplified by the selected primer set.

### 2.5. Optimization of assay with the selected primer pair

The selected primer assay was optimized for various parameters, including the DNA Polymerase, primer annealing temperature, primer concentration, amplification thermocycling temperature ramp rates and extension times. All the optimization reactions were conducted with HCMV and HSV1 DNA.

For comparing the DNA Polymerase enzymes, 10X serial dilutions of HCMV and HSV1 ranging from 10^5^ to 10^2^ genome copies/reaction were amplified in triplicates each with NEB Phusion Hot Start DNA Polymerase and NEB Q5 High Fidelity DNA Polymerase. The reaction conditions were as previously stated with an annealing temperature of 70 °C for both Polymerases.

For annealing temperature optimization, 10X serial dilutions of HCMV and HSV1 ranging from 10^4^ to 10^2^ genome copies/reaction each were amplified in duplicates using the Polymerase selected from the previous step. The experiment was carried out in Bio-Rad CFX96 qPCR instrument for 6 different temperatures ranging from 71.7 °C to 67.4 °C as follows: 71.7 °C, 71.2 °C, 70.2 °C, 69.0 °C, 68.0 °C and 67.4 °C.

Primer concentration experiments were performed with HSV1 by designing a matrix of different primer concentrations ranging from 0.05 to 0.5 μM/reaction for forward and reverse primers. Both asymmetric as well as symmetric primer concentrations were tested. Ramp rates and extension time were also optimized for maximum sensitivity.

### 2.6. Analytical characterization of the assay to assess the limit of quantification, dynamic range and reproducibility

Using the selected primer pair, standard curves were created for eight 10X serial dilutions in triplicates for HCMV and HSV1: 8.45 × 10^7^ to 8.45 × 10^0^ genome copies/reaction for HCMV and 5.23 × 10^7^ to 5.23 × 10^0^ genome copies/reaction for HSV1. For HSV2, seven 10x serial dilutions were used, ranging from 3.45 × 10^6^ to 3.45 × 10^0^ genome copies/reaction. The qPCR standard curves were used to find the linear dynamic range, limit of quantification (LOQ), Ct corresponding to LOQ and the reaction efficiency. The reproducibility of the reaction was found by inter and intra reaction variability through repeat experiments (triplicates for HCMV and HSV2 and duplicates for HSV1).

## 3. Results

### 3.1. Design of universal primers

Of the 51 primer pairs designed, only eight were found to be experimentally specific against human genomic DNA and did not amplify it in initial as well as repeat experiments. However, four of these (p12p2, p12p7, p13p2 and p13p14) formed multiple dimers and/or extraneous PCR products, as observed from the multiple melt curve peaks and multiple bands in gel electrophoresis (Supplementary Fig. 2).

The remaining four of the eight selected primer pairs were tested with HCMV and HSV1. With three of these (p11p2, p11p7 and p11p16), the forward primer, p11, bound to HSV1 at more than one position giving multiple products, as observed by multiple product peaks in the melt curves and bands on gel electrophoresis (Supplementary Fig. 3).

**Fig. 1.**
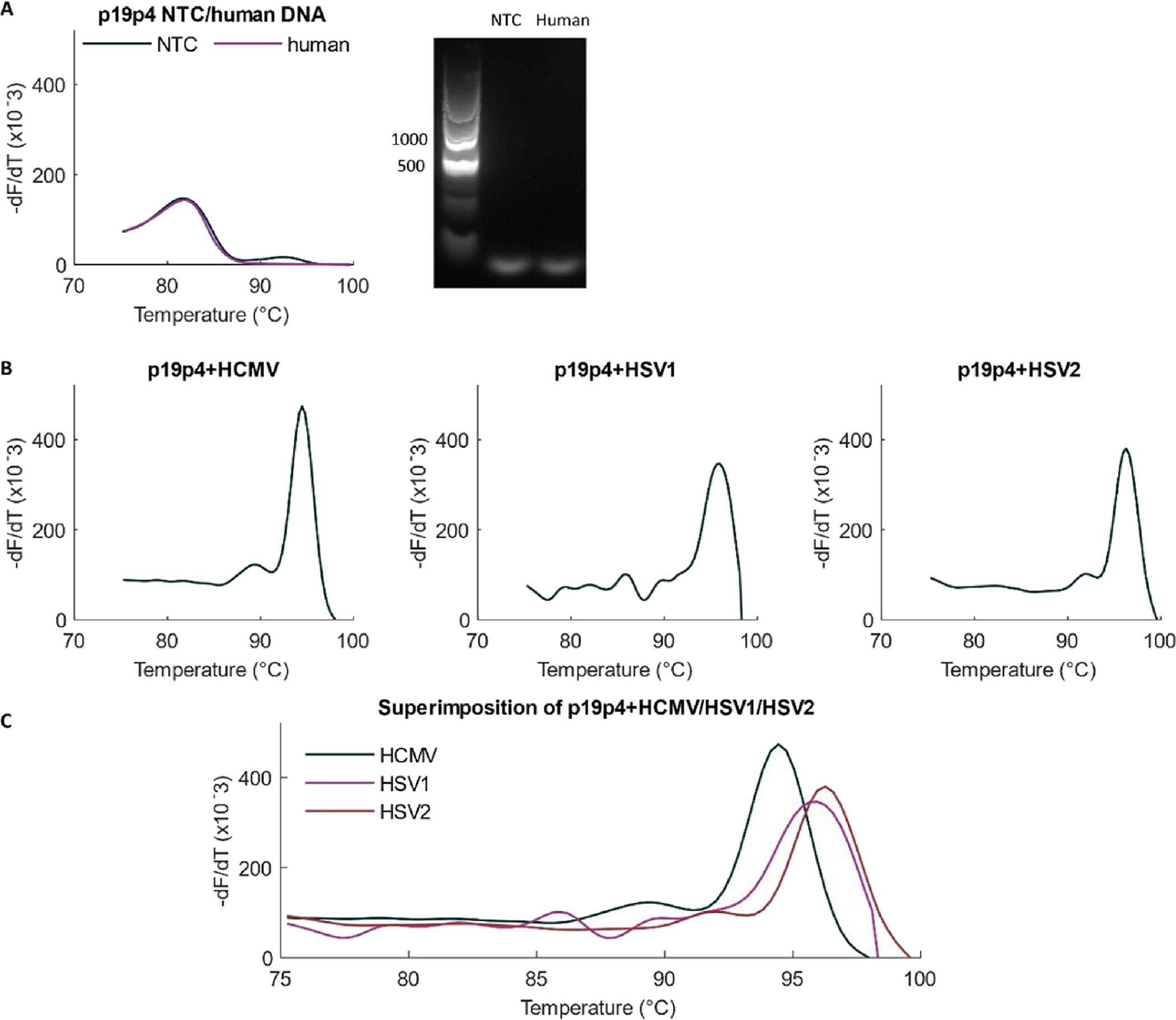
Amplification with primer pair p19p4. (A) The primer pair named p19p4 is specific against human DNA amplification as shown by the alignment for melt curves in NTC and human DNA as template. There was no product melt peak observed with human DNA although the primer forms a primer dimer. (B) Melt curves for each virus with the primer pair p19p4. (C) The melt curves for the three viruses are superimposed together to show their distinct signatures.

The remaining eighth primer set, labeled p19p4, showed specific amplification of all three viruses without amplification of human DNA (Fig. 1). The probe-free specificity can be observed as distinct melting signatures for the three viral amplicons with different average melting temperatures as follows: 94.33 ± 0.32 °C for HCMV, 95.70 ± 0.28 °C for HSV1 and 96.00 ± 0.14 °C for HSV2.

### 3.2. Optimization of the DNA polymerase chemistry of the assay

**Fig. 2.**
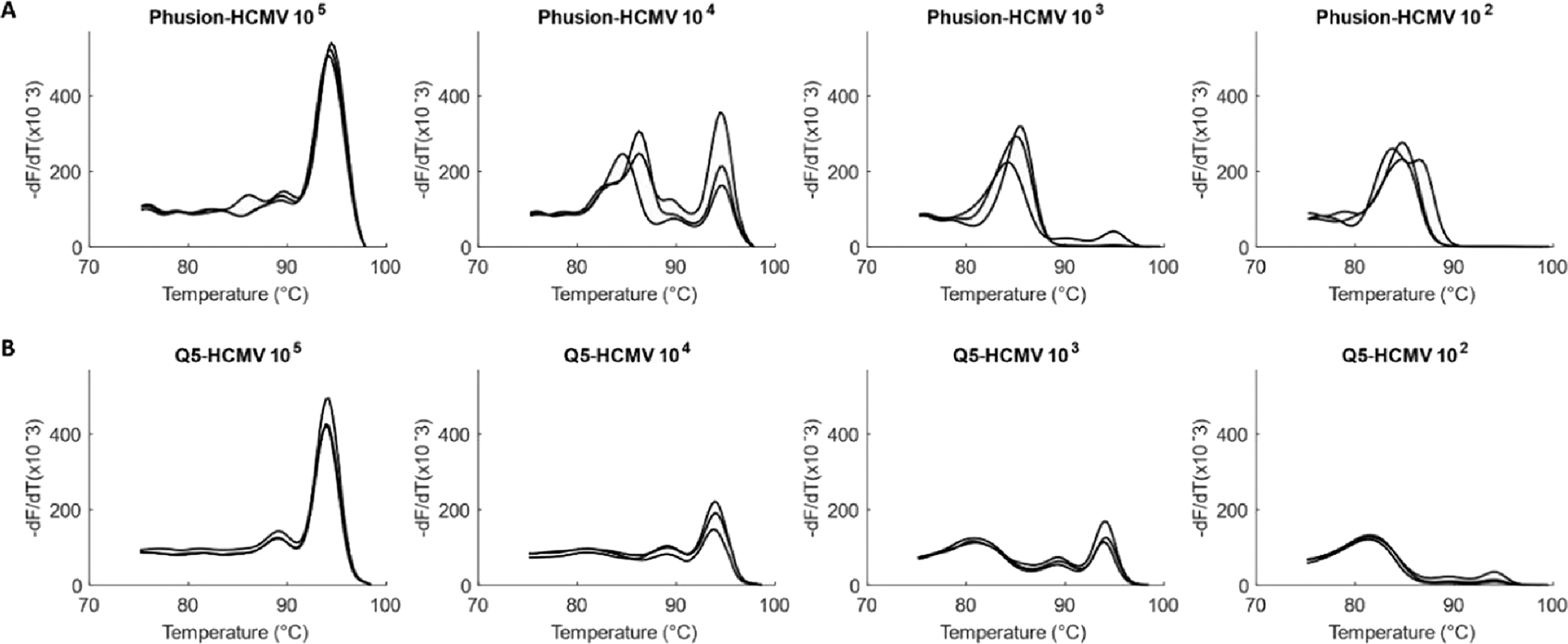
Comparison of Phusion vs Q5 DNA Polymerases for amplification of HCMV. Amplification of serially diluted HCMV (1.53 × 10^5^ to 1.53 ×10^2^ copies/reaction) in triplicates with (A) Phusion-LOD of 1.53 × 10^4^ copies and with (B) Q5-LOD of 1.53 × 10^3^ copies.

**Fig. 3.**
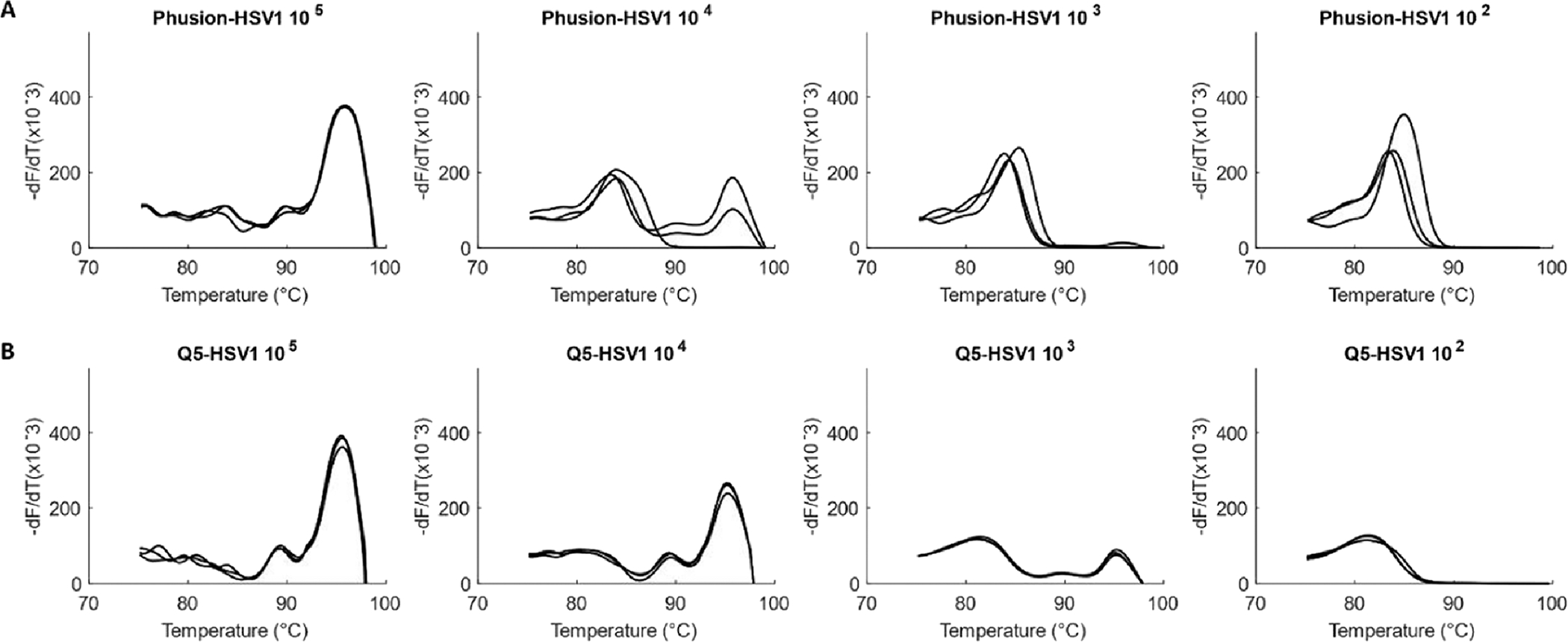
Comparison of Phusion vs Q5 DNA Polymerases for amplification of HSV1. Amplification of serially diluted HSV1 (5.23 × 10^5^ to 5.23 ×10^2^ copies/reaction) in triplicates with (A) Phusion-LOD of 5.23 × 10^4^ copies and with (B) Q5-LOD of 5.23 × 10^3^ copies.

Although initial amplification experiments were performed with Q5 DNA Polymerase, we also wished to test the performance of the assay with High Fidelity NEB Phusion Hot Start DNA Polymerase. With Phusion, the lowest concentration at which product melt peaks were observed, or the limit of detection (LOD), was in the order of 10^4^ viral genome copies/reaction for both HCMV and HSV1– 1.53 × 10^4^ copies/reaction for HCMV and 5.23 × 10^4^ copies/reaction for HSV1. LOD for Q5 was an order lower (better) at 10^3^ viral genome copies/reaction for both viruses (HCMV − 1.53 × 10^3^ copies/reaction, Fig. 2 and HSV1 – 5.23×10^3^ copies/reaction, Fig. 3).

Q5 Polymerase showed better experimental sensitivity for the viral assay and has a higher theoretical fidelity (http://www.neb.com) than Phusion. It was thus chosen as the DNA Polymerase of choice for the viral assay.

### 3.3. Optimization of annealing temperature

**Fig. 4.**
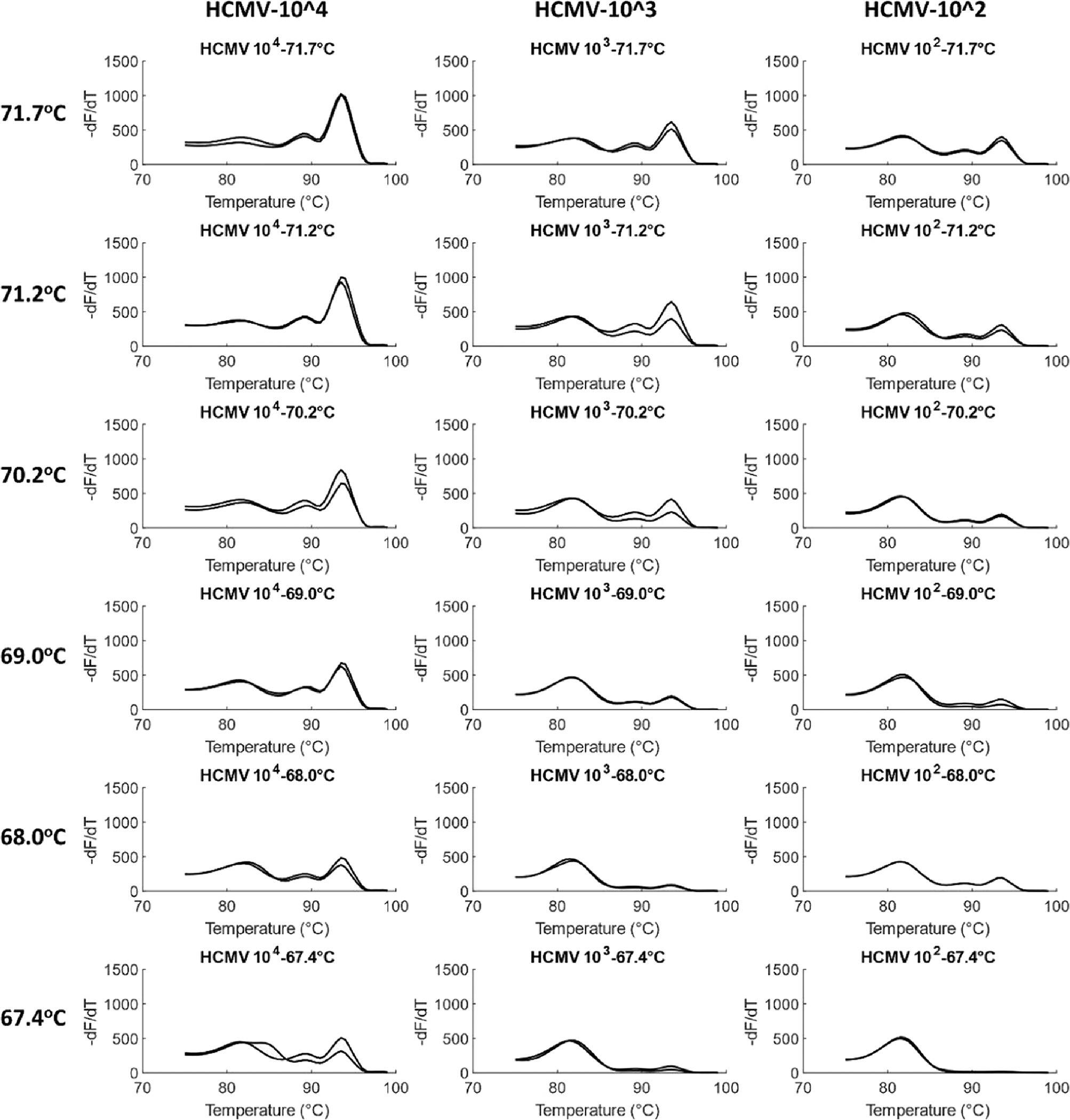
Optimization of annealing temperature in HCMV. Temperature gradient experiments were performed for HCMV in duplicates from 72 °C to 67 °C (rounded off) for a 10× dilution series ranging from 1.53×10^4^ to 1.53×10^2^ genome copies/reaction. Product concentration decreases with decrease in temperature, including for the lowest concentration of 1.53×10^2^ copies/reaction. ~72 °C was thus chosen as the optimum annealing temperature.

**Fig. 5.**
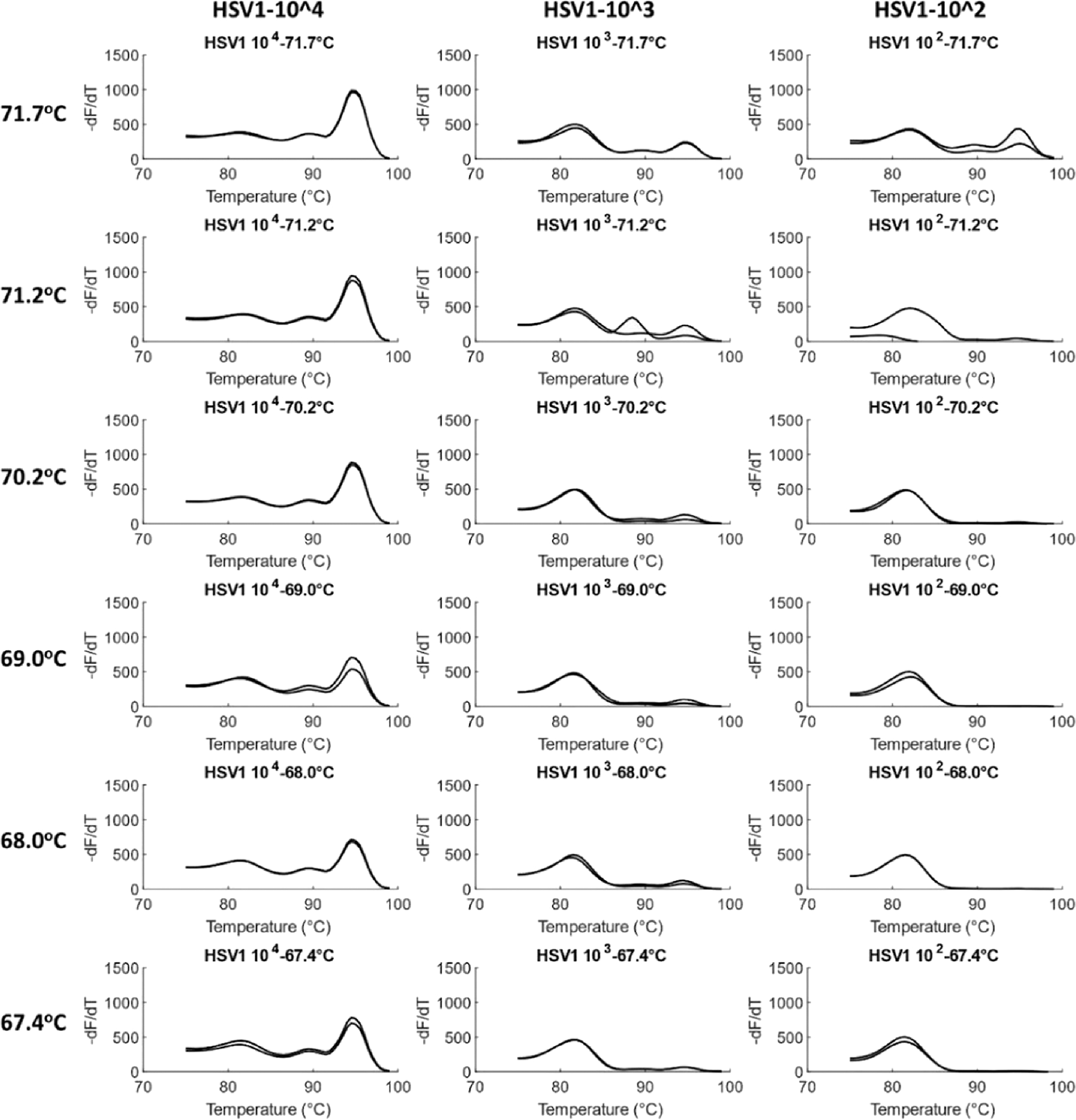
Optimization of annealing temperature in HSV1. Temperature gradient experiments were performed for HSV1 in duplicates from 72 °C to 67 °C (rounded off) for a 10× dilution series ranging from 5.23×10^4^ to 5.23 × 10^2^ genome copies/reaction. As in the case of HCMV, product concentration decreases with decrease in temperature. ~72 °C was thus chosen as the optimum annealing temperature.

A temperature gradient PCR was performed with HCMV and HSV1 to find the optimum annealing temperature. At the highest annealing temperature of 71.7 °C, amplification was observed at all concentrations of the viruses, including at an order of 10^2^ copies/reaction. As the annealing temperature decreased to 67.4 °C in a gradient, amplification at 10^2^ copies/reaction decreased until the melt peak was not observed at all. Simultaneously, at lower temperatures, the occurrence and concentration of primer dimers increased. Thus, 71.7 °C, approximated to 72 °C, was taken as the optimized annealing temperature for the assay. Fig. 4 shows melt curves for HCMV at the gradient temperatures. Similar results were observed for HSV1 (Fig. 5).

Another observation was the improvement of HCMV LOD at 70 °C from 1.53 × 10^3^ copies/reaction in the previous experiment for DNA Polymerase optimization (Fig. 2) to 1.53×10^2^ copies/reaction in the present section. The current experiment was performed in Bio-Rad CFX96 qPCR instrument that was found to have a higher temperature ramp rate during thermocycling. The previous experiment, on the other hand, was performed on ThermoFisher Scientific QuantStudio where a lower ramp rate had been utilized. Therefore, in future experiments, a higher ramp rate was employed in the QuantStudio instrument, which improved the HCMV LOD to 1.53 × 10^2^ or 153 copies.

### 3.4. Optimization of primer concentration

Initial standard curve experiments for both HCMV and HSV1 showed the limit of quantification at least an order higher (poorer) than the limit of detection observed above- for HCMV, LOQ was 8.45 × 10^3^ copies/reaction in standard curve while LOD from optimization experiments (Fig. 4) was 1.53×10^2^ and for HSV1, LOQ was 5.23 × 10^4^ and LOD, 5.23 × 10^2^ (Fig. 5). At concentrations lower than the LOQ, the standard curve showed a long horizontal plateau phase, but the fluorescence did not decrease to zero, as it is expected to in ideal chemistries. This occurs since the primer, p19p4, forms primer dimers even in no template controls (Fig. 1A). At template concentrations below LOQ, free and unutilized primers could be forming dimers, resulting in the fluorescence observed in standard curves below LOQ. Primer dimer bands in gel electrophoresis were also observed at a template concentration of 10^5^ copies/reaction of virus for 0.5 μM of each primer indicating that even at this high template concentration, either some amount of primers was left unutilized or primer dimer formation was completing with product amplification.

**Fig. 6.**
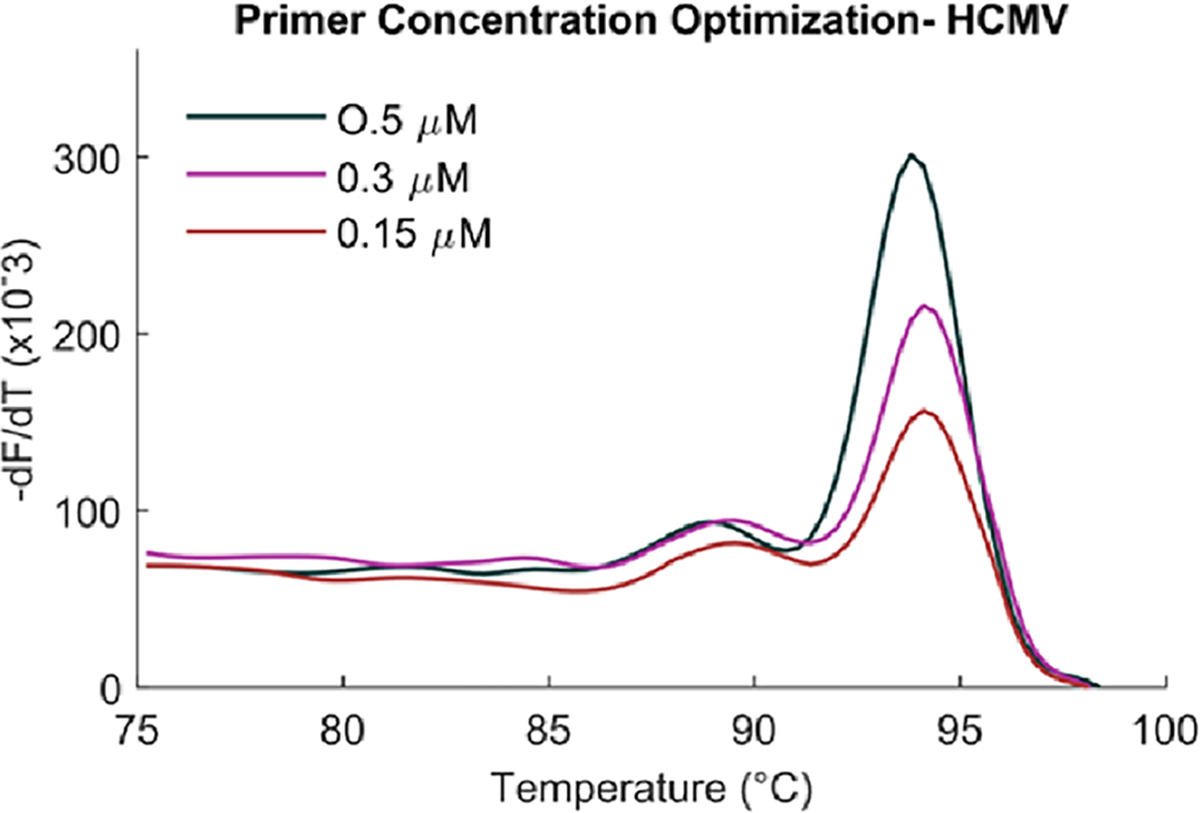
Optimizing primer concentration. Decreasing primer concentrations from 0.5 to 0.15 μM decreased the amount of product formed.

Therefore, an experiment was designed to test if decreasing primer concentration resulted in decreased primer dimer formation such that the product concentration was unaffected. This would also improve the LOQ of the assay. It was observed that at certain lower concentrations of primers, even though primer dimer formation decreased, so did the amount of product formation (Fig. 6). The standard curve LOQ worsened by 2 orders of magnitude at 0.15 μM of each primer as compared to 0.5 μM (not shown). Thus 0.5 μM was considered the optimized concentration for both forward and reverse primers for the assay chemistry.

The optimized probe-free viral assay thus has 0.5 μM/reaction of p19p4 primer and uses Q5 DNA Polymerase. The optimum thermocycling occurs for 30 s annealing at 72 °C and 30 s of extension. The ramp rates were 3 °C/s for initial denaturation and cycle denaturation stages, 2.5 °C/s annealing stage, 3 °C/s for extension stage and final extension after the cycling, and 0.2 °C/s for the final melting stage. The total run time of the assay is 1 h 38 min, with an additional 10 min for initial ramping and final cooling.

A limitation of this optimized assay is the formation of primer dimers even in NTC (Fig. 1A), which affects its analytical characteristics, as discussed below.

### 3.5. Testing the optimized assay with mock samples

The optimized assay was then validated with mock clinical samples to monitor the effect of common clinical inhibitors on its sensitivity. Although the extracted viral DNA was obtained pre-purified from the vendor, blood-extracted human DNA was added to the reaction to simulate a mock clinical sample; an actual viral extraction from blood would result in a similar presence of human DNA and PCR inhibitors. 10X dilution series of HCMV DNA was created from 1.53 × 10^5^ to 1.53 × 10^2^ copies/reaction and ~1.39×10^5^ copies/reaction of extracted human DNA were added to each dilution. Each dilution was amplified in duplicates.

**Fig. 7.**
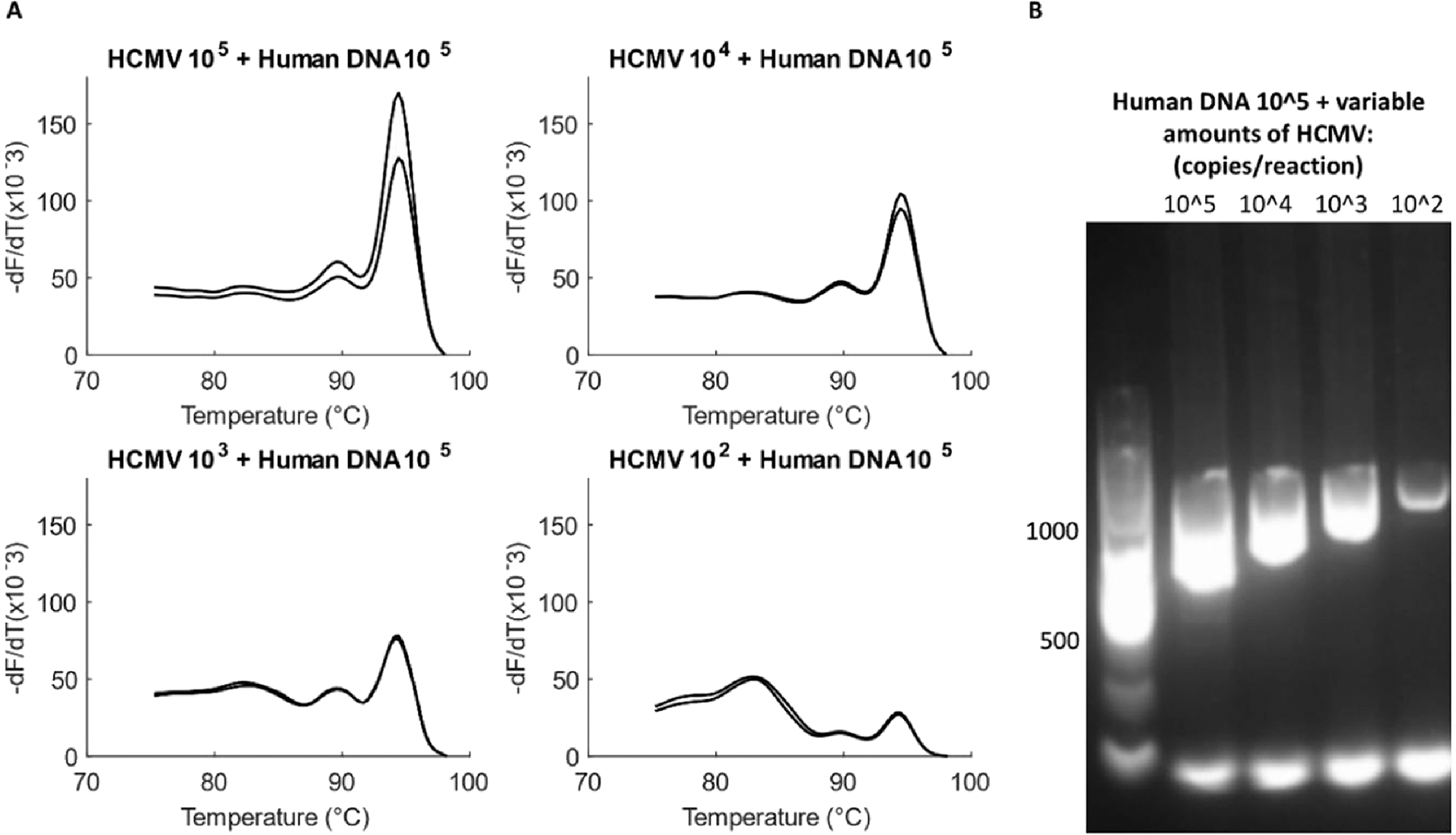
Testing the viral assay with mock samples. (A) Serial dilutions of HCMV (1.53×10^5^ to 1.53 ×10^2^ copies/reaction) added with the same amount of human DNA (1.39×10^5^ copies/reaction), tested in duplicates. HCMV product formation is uninhibited even at the lowest template concentration of 1.53 × 10^2^ copies/reaction. (B) Gel does not show extra bands for amplification of human DNA. Products and primer dimers are both observed at all the tested concentrations of the virus.

The addition of extracted human DNA to the assay did not inhibit the reaction chemistry. The melt curves and gel show similar sensitivity of the HCMV assay as was observed in previous sections (Fig. 7). Specificity of the HCMV assay against human DNA is also underscored by this experiment.

### 3.6. Analytical characterization of the viral assay

**Fig. 8.**
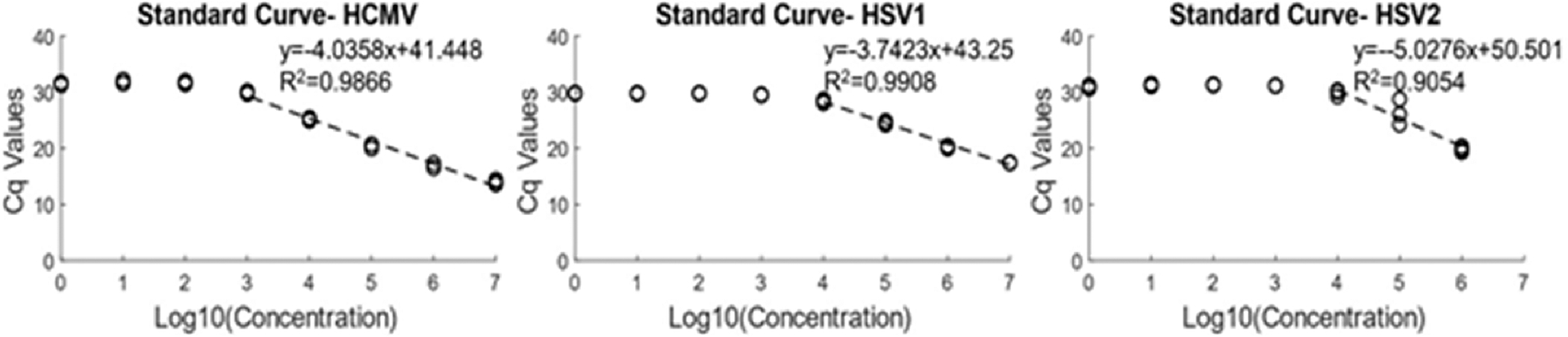
Standard curves for HCMV, HSV1 and HSV2. The quantification region (LOQ) is marked with a dashed line while the plateau region at lower concentrations is due to primer dimers. The serial dilution concentrations are as follows: HCMV-8.45 × 10^7^ to 8.45×10^0^ copies/reaction, HSV1– 5.23 × 10^7^ to 5.23 × 10^0^ copies/reaction and HSV2– 3.45 × 10^6^ to 3.45 × 10^0^ copies/reaction.

Standard curves were created for the three viruses for the analytical characterization of the assay. The LOQ for HCMV is 8.45×10^3^ genome copies/reaction while it is 5.23 × 10^4^ copies/reaction for HSV1 and 3.45×10^4^ copies/reaction for HSV2. Fig. 8 shows the standard curves for the three viruses, with the linear dynamic range marked with a linear fitted line and a plateau phase being observed at lower template concentrations. The linear dynamic range of HSV2 is poorer than the other two viruses since higher concentration of the virus showed inconsistent PCR generally attributed to the presence of inhibitors in the sample. The assay may need to be reoptimized to increase PCR performance for HSV2 or a new HSV2 sample can be ordered to check the validity of the assay. Table 1 shows the characteristics of the assay, including R^2^ values, efficiency and slope in addition to linear dynamic range and LOQ. The efficiency of the assay ranges from 85 % for HSV1, 77 % for HCMV to 58 % for HSV2. The low efficiency could result due to utilization of longer amplicon chemistry or the formation of primer dimers. Our primer design targeted longer amplicons to preferentially detect viable over dead organisms (Mccarty and Atlas, 1993; Immanuel et al., 2020).

Intra and inter-assay variation was found with three repeat standard curve experiments. For HCMV and HSV2, each experiment was with triplicates while for HSV1, one experiment (for determining intra-assay variation) was in triplicates while the other two were duplicates. The inter- and intra-assay coefficient of variation for HCMV and HSV1 were low, ranging from 1.01 % to 2.82 % (intra assay) and from 3.01 % to 5.98 % (inter-assay). The HSV2 experiments had higher intra- and inter-assay variation (Table 2).

## 4. Discussion

This work’s aim was to develop and optimize a probe-free viral assay for HCMV, HSV1 and HSV2. Custom primers were designed for the assay and optimization performed. The optimized assay was found to portray specificity for each of the three viruses with distinct melt curve signatures for each (Fig. 1C). Specificity against human DNA amplification was also observed. The optimized assay has a total turn-around time of < 4 h (2 h for DNA extraction and sample preparation and <2 h for thermocycling and melt imaging). The LOD of the assay is 1.53 × 10^2^ copies/reaction or 10,200 genome copies/ml for HCMV and 5.23×10^2^ copies/reaction or 34,867 genome copies/ml for HSV1 while the LOQ from standard curves was found to be in the orders of 10^3^ copies/reaction for HCMV and 10^4^ for HSV1 (Fig. 8). The HCMV viral load observed in neonates that develop sequelae is ≥ 12,000 copies/ml in whole blood, and plasma levels expected for disseminated HSV in neonates average 15,800,000 copies/ml (~13,000-~400,000,000 copies/ml) (Melvin et al., 2015; Forner et al., 2015b). So, the current LODs of our assay could be useful for detecting clinically relevant viremia in blood. However, HSV DNA load in the plasma of patients with localized infections (skin/eye/mouth or central nervous system) are much lower on average (631 and 158 copies/ml, respectively) and often go undetected in plasma by even the most sensitive PCR assays, suggesting that local sampling is likely necessary to improve molecular diagnostic performance (Melvin et al., 2015).

Thus, a probe-free viral assay was developed that utilizes high resolution melt signatures for specific detection of each pathogenic species, removing the need for sequence-specific probes or nested PCR, and thus increasing the capacity for high throughput detection. The speed and affordability of the assay as compared to viral cultures would allow universal viral screening to be adopted, which is particularly advantageous for cCMV and maternal HSV infections (Cannon et al., 2014; Pinninti and Kimberlin, 2014). Even though clinical manifestations of congenital disseminated viral infections can be relatively nonspecific and overlap with other potentially severe infections, current diagnostic workflows require separate molecular tests. Tests designed to screen for multiple infectious etiologies simultaneously could improve accurate and timely diagnosis for improved treatment and outcomes. Since neonate blood volume restricts the number and volume of samples that can be drawn for diagnostic testing, and therefore the number of tests that can be run, this concept could be even more impactful in this vulnerable patient population.

Notwithstanding the advantages of the assay in clinical neonatal diagnostics, more work needs to be done in addressing the limitations of the assay. The assay shows better performance for HSV1 and CMV than for HSV2, as observed by lower efficiency and poorer linear dynamic range for HSV2. Also, the assay’s sensitivity in a qPCR format is not as sensitive as other more targeted qPCR assays for HSV and HCMV. These limitations likely arose because of the lower amplification efficiencies associated with long amplicon chemistry (HSV1/2 = 741 bp, HCMV = 816 bp) and nucleotide mismatches between the forward primer and HSV1/2. However, long amplicons are expected to preferentially detect viable over dead organisms for potentially better specificity for active infection (Mccarty and Atlas, 1993; Immanuel et al., 2020). The sensitivity would be expected to improve by including a degenerate forward primer and upon translation of the assay to a digital PCR format, where amplification efficiency bias is drastically reduced as a result of endpoint detection (Sinha et al., 2018b). This transition would also enable absolute quantitation and a consensus definition of viral load thresholds that necessitate therapeutic intervention, which is a current limitation of qPCR based nucleic acid tests for viral pathogens (Razonable et al., 2020). Nonetheless, the assay developed herein could enable specific diagnosis and faster clinical decision-making to reduce morbidity from late onset conditions by timely interventions, thus reducing overall healthcare costs.

A future aim for the viral assay would be its multiplexing with our lab’s bacterial and fungal sepsis assays to obtain a single multiplexed diagnostic panel for different pathogenic etiologies in cases of clinically ambiguous symptoms. The probe-free chemistry of the assay would allow easier multiplexing where the species level specificity would be attained through melt signatures. Translation of the multiplexed assay to our lab’s digital molecular technology (digital PCR or dPCR) would further increase its sensitivity to single genome level along with broad based detection, employing the lab’s machine learning algorithm to “learn” unique melt signatures of a multitude of amplicons. This would also result in the usage of a smaller sample volume than traditional PCR and enable absolute quantification of viral loads.

## Supplementary Material

Supplemental Data

## Figures and Tables

**Table 1:** Characteristics of the viral assay for HCMV, HSV1 and HSV2 amplification.

	Linear Dynamic Range	LOQ (copies/reaction)	R^2^	Efficiency (%)	Slope	Y intercept	Mean Cq at LOQ

HCMV	10^7–10^3	10^3	0.987	76.92	−4.04	41.45	30
HSV1	10^7–10^4	10^4	0.991	85.022	−3.74	43.25	28
HSV2	10^6–10^4	10^4	0.905	58.089	−5.03	50.50	30

**Table 2:** Inter and intra-assay variation for the three viruses.

	HCMV		HSV1		HSV2	

	**Mean Ct**	**%CV**	**Mean Ct**	**%CV**	**Mean Ct**	**%CV**
**Intra-assay**						
1x10^6	13.95	2.63	17.41	1.17	19.84	2.46
1x10^5	20.45	1.96	24.59	1.37	26.35	8.61
1x10^4	25.20	1.01	28.43	1.18	29.90	1.92
**Inter-assay**						
1x10^6	13.46	3.12	18.57	1.64	22.38	11.69
1x10^5	19.73	3.29	24.39	3.70	25.90	5.11
1x10^4	24.46	3.01	29.39	3.93	29.74	1.35
