# Supplementary figures and images for: A broad-based probe-free qPCR assay for detection and discrimination of three human herpes viruses
Source: J Virol Methods. Author manuscript; Available in PMC 2024 Jun 13. (PMC11175599; doi:10.1016/j.jviromet.2023.114824)

**Supplementary Figure 1**


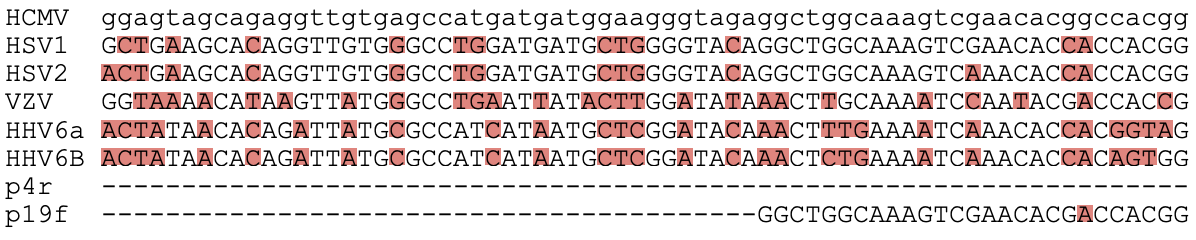


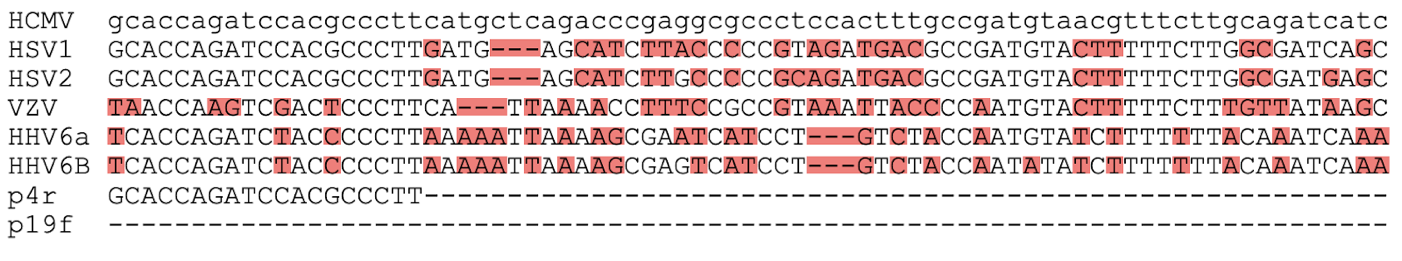


**Supplementary Figure 2**


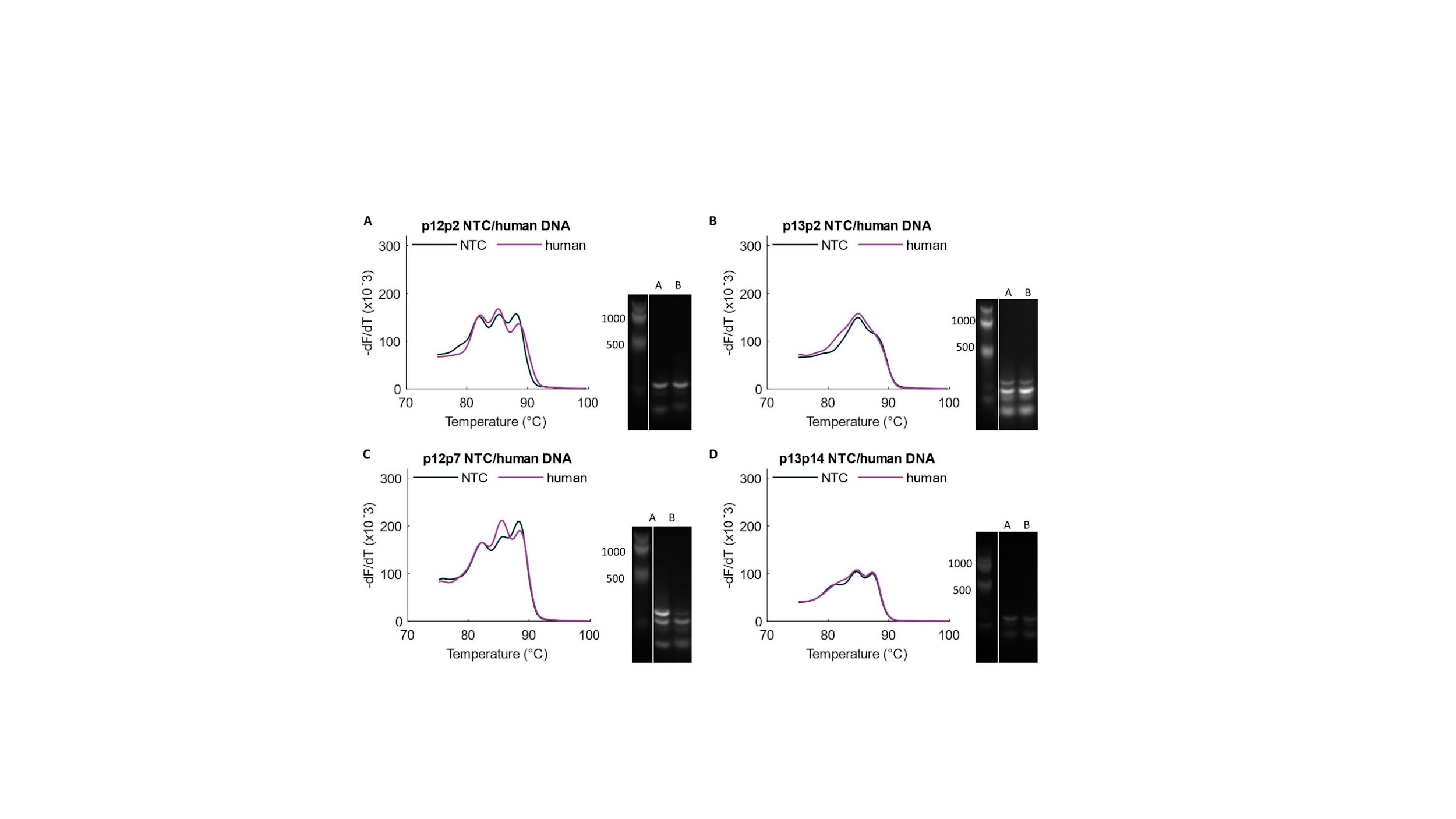


**Supplementary Figure 3**


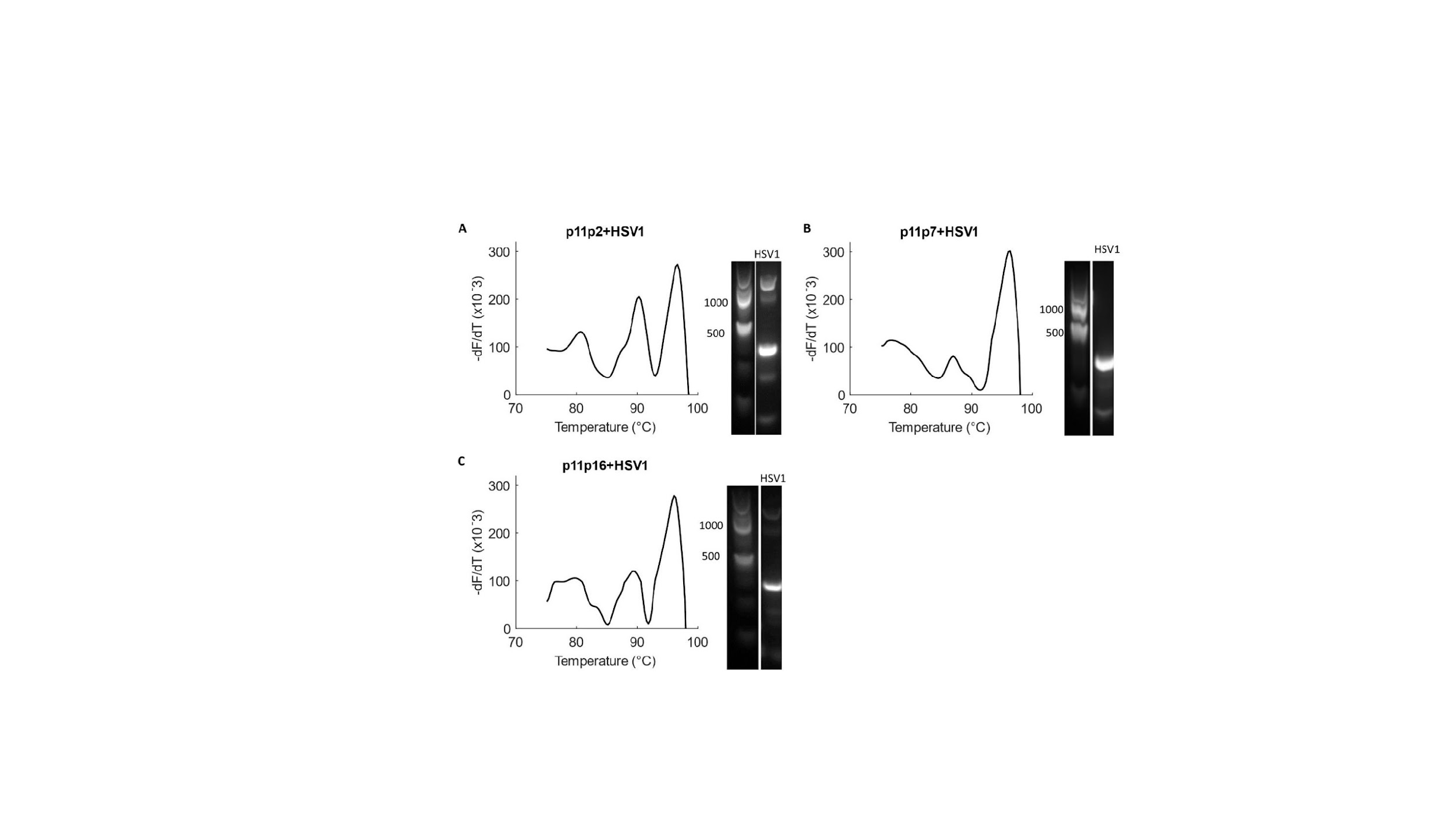

Supplement: Supplemental Data [file NIHMS1994773-supplement-Supplemental_Data.docx]
